# Elastomeric Porous Poly(glycerol sebacate) Methacrylate (PGSm) Microspheres as 3D Scaffolds for Chondrocyte Culture and Cartilage Tissue Engineering

**DOI:** 10.3390/ijms241310445

**Published:** 2023-06-21

**Authors:** Dharaminder Singh, Sarah Lindsay, Shruti Gurbaxani, Aileen Crawford, Frederik Claeyssens

**Affiliations:** 1 Kroto Research Institute, Department of Materials Science and Engineering, The University of Sheffield, Broad Lane, Sheffield S3 7HQ, UK; 2 School of Clinical Dentistry, The University of Sheffield, Claremont Crescent, Sheffield S10 2TN, UK; 3 Insigneo Institute for in Silico Medicine, The University of Sheffield, Sheffield S10 2TN, UK

**Keywords:** biomaterial, scaffolds, biodegradable, tissue engineering, polymer, elastomer, cartilage, microspheres, polyHIPE, emulsion templating, PGSm

## Abstract

Cartilage defects can be difficult to treat; therefore, tissue engineering of cartilage is emerging as a promising potential therapy. One interesting area of research explores the delivery of cells to the cartilage defect via scaffold-based cell delivery vehicles and microsurgery. This study explores the use of novel poly(glycerol sebacate) methacrylate (PGSm)-polymerised high internal phase emulsion (polyHIPE) microspheres as scaffolds with embedded cells for cartilage tissue engineering. Porous microsphere scaffolds (100 µm–1 mm diameter) were produced from emulsions consisting of water and a methacrylate-based photocurable resin of poly(glycerol sebacate). These resins were used in conjunction with a T-junction fluidic device and an ultraviolet (UV) curing lamp to produce porous microspheres with a tuneable size. This technique produced biodegradable PGSm microspheres with similar mechanical properties to cartilage. We further explore these microspheres as scaffolds for three-dimensional culture of chondrocytes. The microspheres proved to be very efficient scaffolds for primary chondrocyte culture and were covered by a dense extracellular matrix (ECM) network during the culture period, creating a tissue disk. The presence of glycosaminoglycans (GAGs) and collagen-II was confirmed, highlighting the utility of the PGSm microspheres as a delivery vehicle for chondrocytes. A number of imaging techniques were utilised to analyse the tissue disk and develop methodologies to characterise the resultant tissue. This study highlights the utility of porous PGSm microspheres for cartilage tissue engineering.

## 1. Introduction

The limited spontaneous regeneration of cartilage has driven research toward tissue engineering to repair injuries to this tissue. Therefore, tissue-engineered cartilage is a widely researched area for the repair of joint damage as well as the repair of nasal and auricular cartilage [1,2]. Cartilage is a highly specialised, dense connective tissue, serving to support and give shape to softer tissues, provide a low friction area for free articulation of joints and provide a model for bone formation [3]. Hyaline cartilage, the most common type of cartilage, is often found as articular cartilage at the ends of diarthrodial joints. Damage to articular cartilage through injury or pathology often leads to osteoarthritis, causing joint pain, deterioration of the joint leading to a loss of function and ultimately, a total joint replacement. Articular cartilage is avascular, with a lack of nerves and lymphatic system, limiting its capacity for repair [4]. Total joint replacements should remain a last resort; however, all other surgical techniques are currently ineffective at stopping the long-term destruction of the joint, which is why innovative tissue-engineered approaches for developing and regenerating articular cartilage have been explored [4,5]. Cartilage regeneration techniques currently being explored/in use can generally be characterised by: microfracture and bone marrow stimulation, autologous chondrocyte implantation and matrix-induced autologous chondrocyte implantation (MACI) [5].

Briefly, microfracture utilises the body’s ability to repair itself by creating a wound site, allowing MSCs to migrate from the bone marrow and initiate wound repair [6,7,8]. Whilst fresh cartilage formation is stimulated by microfracture, it often produces inferior fibrocartilage, as opposed to the preferred hyaline cartilage. Studies have shown that this mechanically inferior cartilage does not perform in the long term and begins to break down at 18 months [5,7,9,10]. Autologous chondrocyte implantation utilises an in vitro culture of the patients’ autologous chondrocytes to stimulate the regeneration of fresh cartilage. An initial surgery is required to remove a healthy full-thickness biopsy for the isolation, culture and expansion of autologous chondrocytes in vitro. Cells are implanted into the damaged cartilage area in a follow-up surgery and covered by a periosteal patch or collagen membrane. ACI studies appear effective, with one study stating that 10–20 years after surgery, 74% of the patients reported their status as better or the same as previous years [11]. Periosteal patches have reported hypertrophy, mechanical problems and joint revisions, whereas collagen patches may be limited in their use by the regulatory bodies [12]. Cell distribution irregularities and the potential for cell leakage have led to studies incorporating 3D scaffolds and matrices [12,13]. Scaffolds as cell carriers for tissue-engineered cartilage may better distribute cells, fill cartilage defects more effectively, allow for quicker recovery due to scaffold stability, fewer donor site complications and benefit from a simpler surgical procedure [5]. Whilst 2D monolayer cultures of chondrocytes often dedifferentiate and progress towards a fibroblast phenotype to produce fibrocartilage, studies show that 3D cultures have a more potent chondrogenic potential [14]. Therefore, through the implantation of a 3D environment, it may be possible to limit the dedifferentiation of the chondrocytes and stimulate more hyaline-like cartilage. MACI utilises a bilayer scaffold from porcine type I/III collagen, with smooth, dense fibres on one side, and rough, wide-spaced fibres on the other [12]. The initial surgery collects autologous tissue, from which chondrocytes are isolated, expanded in the lab and seeded onto the scaffold. Seeded scaffolds are attached, in a secondary surgery, to the chondral base with fibrin glue, negating the need for a membrane cover. Results from MACI are largely positive, with lower incidences of hypertrophy reported, and it was noted that MACI is quicker to perform surgically [12]. Whilst MACI offers many potential benefits, the superiority over other techniques remains unclear. Hyaluronic acid- and fibrin-based scaffolds are other materials explored in a similar procedure to MACI, with similar results [5].

Tissue-engineered cartilage research focusses on the isolation of cells, the culture of these cells seeded onto a biomaterial scaffold, or within a liquid precursor, and the eventual implantation of the scaffold into an injury site. Despite the instability and loss of phenotype in monolayer cultures, chondrocytes remain the obvious choice of cells to use, with chondrocytes being the only cell type in cartilage with its natural ability to produce a cartilage ECM. Scaffolds synthesised from natural biomaterials offer the benefit of increased biocompatibility, however, synthetic biomaterials allow for more versatility and reproducibility in scaffold design. Natural scaffolds are often protein- or polysaccharide-based, whereas synthetic scaffolds are often synthesised from soft polymers. For example, BioSeed-C, a product from BioTissue Technologies (Freiburg, Germany), is a synthetic polymer graft seeded with autologous chondrocytes. This has shown some success, reporting improved clinical scores in humans [15,16].

An ideal scaffold material is compatible with the tissue it is implanted in, i.e., supports good cell interaction, including adhesion, growth and differentiation, and has a low local inflammatory response. Ideally, the material offers a conducive 3D microenvironment, allows for diffusion, degrades in a controlled manner and has similar mechanical properties to the native tissue. Hollister’s research group explored the use of additive manufactured poly(glycerol sebacate) (PGS) scaffolds via fused-filament fabrication for cartilage repair in 2010 and 2015, and concluded that PGS is a suitable candidate material for cartilage tissue engineering [17,18]. Additional to standard PGS, polyethylene-glycol (PEG)-modified PGS (PGS-co-PEG) [19] and PGS composites with bio-glass [20] have been proposed for cartilage tissue engineering. PGS has been used to make scaffolds using various production techniques; for example, electrospinning was used to produce aligned nanoscale coaxial PGS/PCL fibres with the inclusion of Kartogenin (a chondrogenic and chondroprotective agent) [21]. These fibre scaffolds promoted the chondrogenic differentiation of human bone marrow stromal cells (hBMSCs). Additionally, a two-layer PGS-co-PEG/mesoporous bio-glass scaffold was proposed for cartilage tissue engineering by Lin et al. [22]. This scaffold showed regeneration of a well-integrated articular hyaline cartilage and subchondral bone within 12 weeks in a New Zealand white rabbit full-thickness osteochondral defect.

In this study, we explore poly(glycerol sebacate methacrylate) (PGSm), a photocurable formulation of PGS. This material was first reported in 2018 by Singh et al. and has been used to explore 3D-printed conduits for peripheral nerve repair in vivo [23]. In short, this polymer is synthesised in two steps: (i) via condensation polymerisation of a 1:1 ratio of glycerol to sebacic acid and (ii) via methacrylation of the remaining hydroxyl end-groups. Both glycerol and sebacic acid are natural materials that are FDA-approved for implant use, and the methacrylation renders the polymer photocurable. PGS-based scaffolds have been suggested for a number of tissue engineering applications, including cardiac patches, retinal transplantation and neuronal repair [23].

The aim of this study is to explore the potential of PGSm microspheres as injectable cell (chondrocyte) delivery vehicles for articular cartilage repair and to determine imaging/analysis techniques. The delivery of the cell-laden microspheres could be achieved via a keyhole procedure similar to needle arthroscopy, where an arthroscopy cannula could be used to deliver the microspheres, as long as the diameter of the microspheres is smaller than the internal diameter of the arthroscopy cannula (typically >2 mm).

PGSm has many characteristics beneficial for cartilage tissue repair, including biocompatibility, biodegradation, appropriate mechanical properties and the ability to photo-crosslink into complex 3D structures. This study demonstrates the development of PGSm into a polyHIPE (high internal phase emulsion) and into porous polyHIPE microspheres for cartilage tissue engineering. PolyHIPE microspheres with tuneable dimensions and interconnected pores were used as a 3D scaffold for chondrocytes. Chondrocytes embedded in these individual microspheres produced the ECM, linking the microspheres together, creating one large 3D scaffold. This study sets out to investigate alternative and more effective means of cartilage tissue engineering through the synthesis of tuneable porous polyHIPE PGSm microspheres, the proof-of-concept culture of embedded chondrocytes in vitro, through mechanical and biological assessment and through image analysis of the formed tissue.

## 2. Results

**Figure 1:**
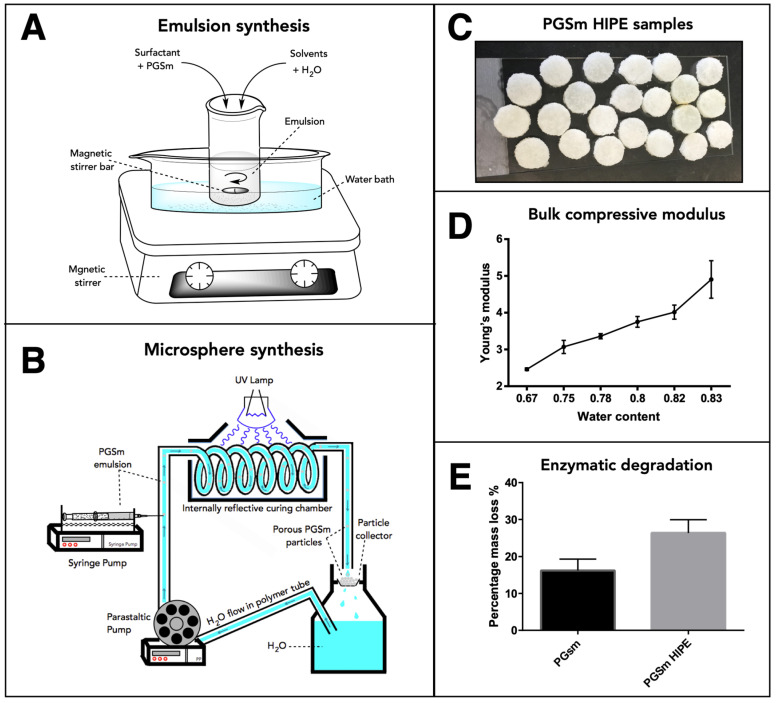
Schematic drawing of the experimental setup to produce (**A**) the high internal phase emulsions and (**B**) the microspheres. (**C**) PGSm polyHIPE samples to be used for mechanical testing and enzymatic degradation studies. Results from the mechanical testing (**D**) and enzymatic degradation studies (**E**) are shown (standard deviation error bars, *n* = 3).

PGSm polyHIPEs were synthesised and the bulk material was analysed. The compressive mechanical modulus was determined for the dry samples and increased in line with the water:polymer ratio of the emulsion (Figure 1D). The bulk material had a compressive modulus value of 4.01 MPa with an emulsion water:polymer ratio of 0.82. Enzymatic degradation studies were performed on PGSm and PGSm polyHIPE samples. At day 9, PGSm samples showed a percentage mass loss of 17%, whereas PGSm polyHIPE samples showed a percentage mass loss of 27%.

**Figure 2:**
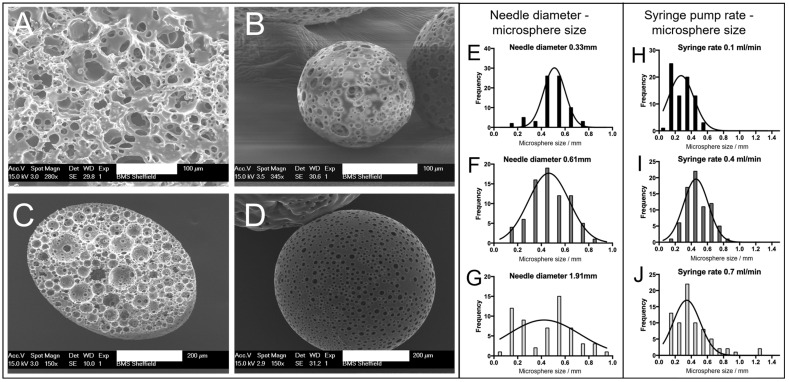
SEM images of the crosslinked emulsion and microspheres. (**A**) The bulk emulsion when fully crosslinked and dried, scale bar 100 µm. (**B**,**D**) Synthesised microspheres, scale bar 100 µm and 200 µm, respectively. (**C**) A cross-section of a microsphere showing a highly porous interior, scale bar 200 µm. Histograms showing the effects of two experimental variables on the produced microsphere size. Histograms in (**E**–**G**) demonstrate the effect of changing the syringe needle diameter (0.33 mm, 0.61 mm, 1.91 mm) on the microsphere size. (**H**–**J**) The effect of changing the syringe pump rate (0.1 mL/min, 0.4 mL/min, 0.7 mL/min, needle diameter 0.61) on the microsphere size. All histograms were produced using SEM image analysis, whereby 75 individual microsphere diameters were measured using Image J (version 1.53t, NIH). All histograms were fitted with a Gaussian distribution curve.

The PGSm HIPE (Figure 1A) was used to create porous microspheres using the microsphere synthesis setup (Figure 1B). SEM images were taken of the resulting bulk poly-HIPE when it was fully crosslinked and dried (Figure 2A). A highly porous network was observed, with interconnected pores between the voids where water droplets were removed upon drying. The SEM images (Figure 2B,D) showed microspheres of differing sizes (~150 and ~500 µm) produced from the emulsion using the microsphere synthesis setup, indicating a porous exterior. The microspheres also exhibited a porous interior, which was confirmed from a microsphere that was sectioned (Figure 2C). These images support that porous PGSm microspheres could be rapidly produced, exhibiting continuous porosity, in a reproducible manner.

The polyHIPE solution was Injected via a syringe in a carrier fluid (water) with a set injection speed. Increasing the syringe needle size when synthesizing the microspheres had a direct effect on the microsphere size distribution (Figure 2E–G). From the Gaussian curve fit to the three histograms (Figure 2E–G), it is evident that the bell curve peaks were at a similar value with all three needle diameters (30 G, 23 G and 15 G), suggesting that the average microsphere size did not notably change when changing the needle diameter. The spread of microsphere sizes within a batch did, however, change with the needle diameter, with the largest spread of microsphere sizes at the largest needle diameter. It can, therefore, be noted that a small needle diameter may be more appropriate for the synthesis of microspheres of a homogenous size, as can be seen in Figure 2E, where the microsphere sizes were mainly between 0.45 mm and 0.55 mm. Figure 2G shows a wide microsphere size distribution, from ~0.1 to 1 mm microspheres. The distribution of microspheres in Figure 2F (23 G syringe needle) shows an intermediate distributed spread of microsphere sizes, with the graph conforming to a recognizable Gaussian distribution between 0.2 and 0.8 mm microsphere sizes (average microsphere size 465 ± 158 µm, SD, *n = 75*). The histograms (Figure 2H–J) show the effect of altering the syringe pump rate on the microsphere size. The histograms indicate that the microsphere size distribution broadened with the increasing syringe pump speed, as seen from Figure 2H–J. This can be seen by the largest group of microspheres in each, clearly increasing in size from 0.6 mm in Figure 2H to 1.2 mm in Figure 2J, while the peak of the Gaussian curve fit did not notably change.

**Figure 3:**
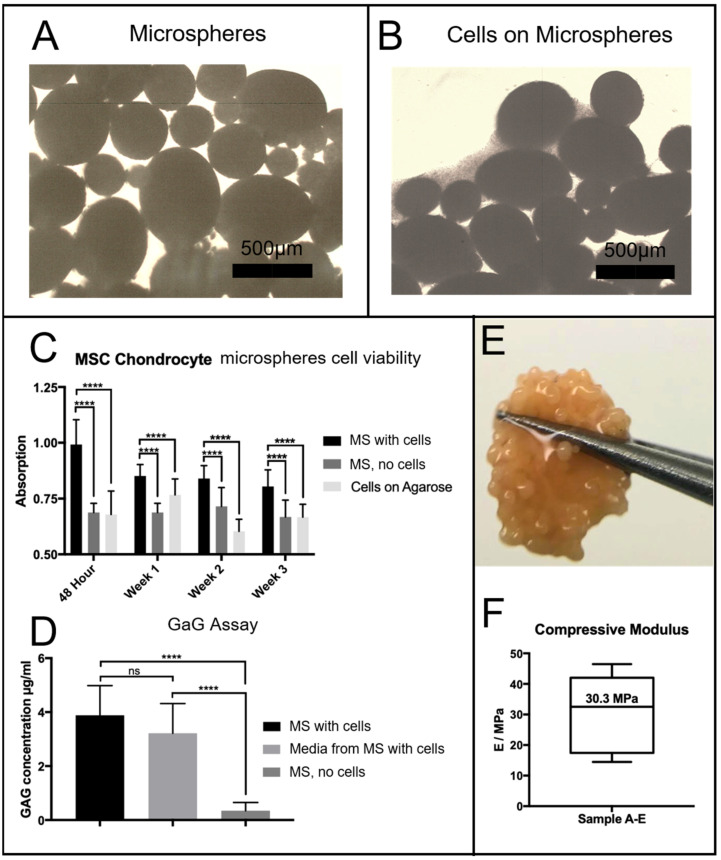
Light microscope images of (**A**) PGSm microspheres without cells and (**B**) PGSm microspheres with cells (scale bar 500 µm) 24 h after seeding. (**C**) Resazurin metabolic assay of the microspheres (MS) seeded with cells, compared to the signal of a negative control (cell-free microspheres (MS)) and the cells seeded onto agarose. This study was performed at 48 h, and on days 7, 14 and 21. A two-way ANOVA with Tukey’s post hoc test showed a statistically significant difference between the seeded microspheres and other variables at each time point (****, *p* ≤ 0.0001, *n* = 3). (**D**) A DMB assay for GaGs was performed after three weeks. This was performed on microspheres (MS) seeded with cells, medium from microspheres (MS) and cell-free microspheres (MS). A one-way ANOVA with Tukey’s post hoc test showed a statistically significant difference between both microspheres with cells and medium from the microspheres with cells when compared with microspheres without cells (****, *p* ≤ 0.0001). There was no statistically significant difference (ns) between the microspheres with cells and media from the microspheres with cells. (**E**) A photograph of the microspheres following 3 weeks of culture: extracellular matrix/collagen had grown across and through the microspheres, creating a strong disk, as seen held between tweezers (approximate disk diameter 5 mm). (**F**) Compressive modulus of the microspheres/microsphere disk after 3 weeks in culture, with a mean compressive modulus of 30.3 MPa (*n* = 5).

Chondrocytes were seeded onto microspheres using the protocols described above. After 24 h, chondrocyte cells had begun to attach to the PGSm microspheres. From the microscope images (Figure 3), chondrocytes appeared attached to microspheres seeded with chondrocytes (Figure 3B). Microspheres not seeded with chondrocytes clearly had no cells/cellular material surrounding them (Figure 3A).

Chondrocyte cells were seeded onto the microspheres and cultured for a period of three weeks. Agarose-coated wells contained either porous PGSm microspheres seeded with chondrocytes, plain agarose-coated wells seeded with chondrocytes or porous PGSm microspheres without cells (cell-free microspheres). Well plates were placed on an orbital shaker for a 48 h seeding period, allowing maximum attachment opportunities for the chondrocytes to attach to the microspheres. Following the cell-seeding period, cell attachment was visible on the light microscope (Figure 3B). A resazurin cell viability assay was performed at 48 h, week 1, week 2 and week 3 (Figure 3C). From the 48 h time point, there was a significant difference between the microspheres with cells and the two cell-free controls, indicating metabolic activity of the chondrocytes. This pattern occurred over each of the time points over the three weeks, indicating cell survival on the microspheres for the duration of the experiment. A Dimethylmethylene Blue (DMB) assay determined the presence of GaGs, [24] a marker for the maturity of the chondrocyte culture (Figure 3D). Results of the DMB assay at week 3 showed a significant presence of GaGs in both the chondrocyte-seeded microspheres and the media from the chondrocyte-seeded microspheres, when compared with the microspheres without cells. By the third week, cells within the microspheres had produced enough ECM to aggregate and form a microsphere tissue disk. The digital photograph (Figure 3E) shows the microsphere tissue disk, robust enough to be handled without damage. The microspheres without cells seeded remained as individual microspheres over the three weeks. Microsphere tissue disks were compressed to provide mechanical compressive data and a stiffness value. The average compressive modulus value of tissue microsphere disks was observed to be 30.3 MPa (Figure 3F), while the stiffness of the polyHIPE scaffolds measured 4 MPa. The microsphere tissue disk was compressed in an unconfined manner and the disk was observed to laterally spread out during compression. Additionally, the microsphere tissue disks remained whole throughout the compression, highlighting the strength of the newly formed tissue disk.

**Figure 4:**
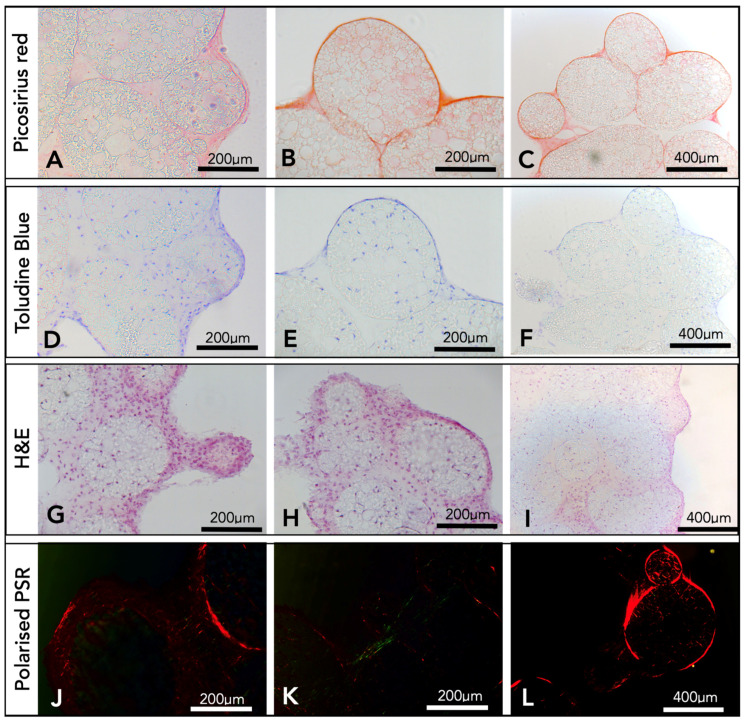
Histological results from microspheres seeded with chondrocytes after three weeks. The first two columns have 200 µm scale bars and the end column has a 400 µm scale bar. The first row (**A**–**C**) shows results from a Picro sirius red stain. The second row (**D**–**F**) shows results of the toluidine blue stain, with the stain showing as deep blue and purple. The third row (**G**–**I**) shows results from an H&E stain, and these three rows were imaged using an upright light microscope. The final row (**J**–**L**) represents the Picro sirius red stain, imaged using an Olympus BX61 upright microscope, setup using a rotatable polariser under the condenser.

After a period of three weeks, the microsphere tissue disks were analysed by histology. Picro sirius red staining produced images with red collagen fibrils, both surrounding and within the microspheres (Figure 4A–C). Toluidine blue (Figure 4D–F) stained the nucleic acid from chondrocyte cells blue, and cells were observed throughout the porous interior network of the microspheres and within the surrounding ECM. There was also evidence of the stain showing areas of deep purple (Figure 4D), an indicator for the presence of GaGs. Haematoxylin and Eosin histology results (Figure 4G–I) confirmed the presence of cells and the ECM surrounding the microspheres and throughout the inside of the porous microspheres. Picro sirius red-stained samples were imaged using a polarised light microscope, and fibres of both red and green were observed throughout and surrounding the porous PGSm microspheres (Figure 4J–L). The histological results overall showed that microspheres have been clustered together with fibres of collagen and indicated the presence of components found within the cartilage ECM.

**Figure 5:**
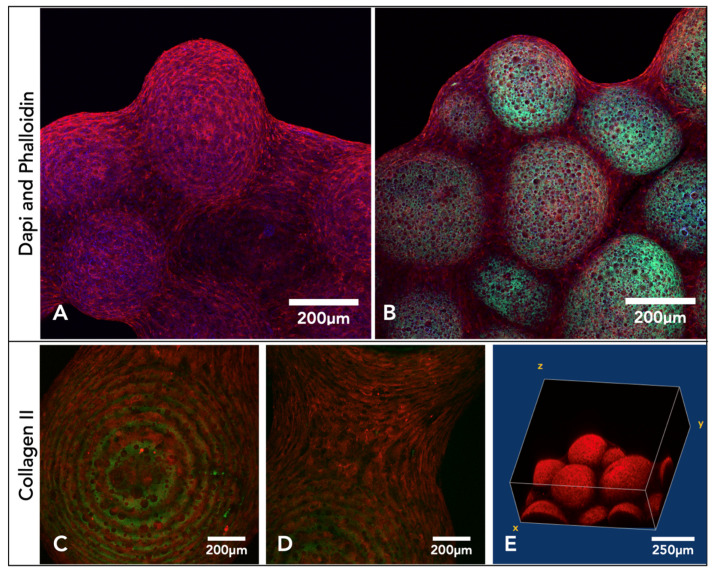
Confocal microscopy images compiled from Z-stacks of microsphere tissue disks labelled with either DAPI and phalloidin or a collagen-II antibody after three weeks of culture with chondrocytes. (**A**,**B**) Microsphere disk labelled with TRITC phalloidin (red) and DAPI (blue), and the porous polymer microspheres also auto-fluoresced (green). (**C**–**E**) Confocal images of the microsphere tissue disks, IHC-labelled with anti-collagen-II antibody and secondary antibody Alexa 546 (red). (**E**) Slices from the Z-stack compiled into a 3D image.

After three weeks in culture, microsphere tissue discs were immunohistochemically labelled and imaged on a confocal microscope, collecting z-stack micrographs. DAPI- and phalloidin TRITC-stained (nucleic acid/cell nuclei and F-actin/cytoskeleton) samples show the microsphere tissue disk covered in the ECM created by the chondrocytes (Figure 5A,B). Microsphere tissue disks were also labelled with anti-collagen-II antibody, and the images indicate a clear presence of collagen-II within the microsphere tissue disk (Figure 5C–E).

**Figure 6:**
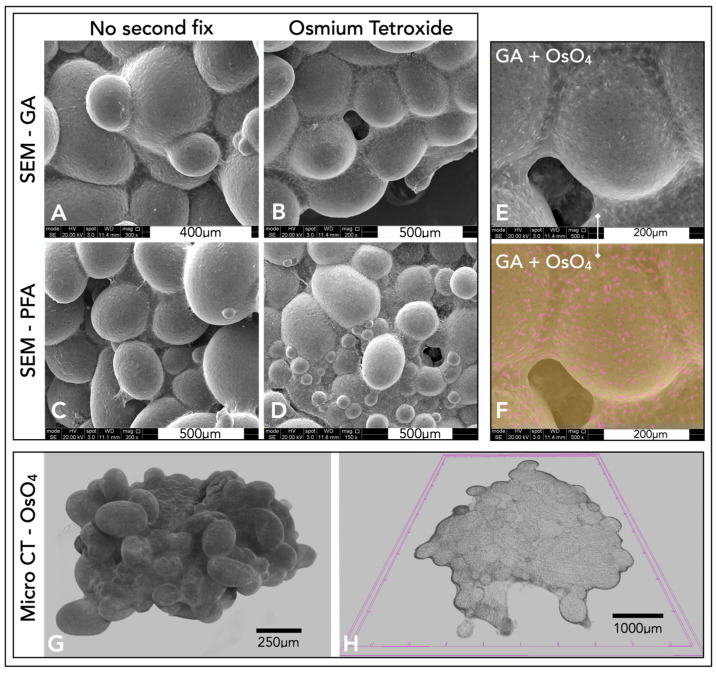
(**A**–**D**) SEM images of microsphere tissue disks prepared with different techniques. (**G**,**H**) Micro-CT models of the microsphere tissue disk. Images (**A**,**B**) were fixed in glutaraldehyde and (**C**,**D**) were fixed in paraformaldehyde. Images (**A**,**C**) had no secondary fix, whereas images (**B**,**D**) had a secondary fix in 2% aqueous osmium tetroxide for two hours. (**E**) A higher-magnification SEM image of the microsphere disk with a glutaraldehyde and osmium fix (same as (**D**)). (**F**) A false colour representation of (**E**). (**G**,**H**) Micro-CT representations of the microsphere tissue disks fixed in glutaraldehyde and osmium tetroxide. (**G**) A three-dimensional representation of the disk. (**H**) A slice within this polymer disk.

Different fixation techniques were explored to understand the optimal method for SEM imaging (Figure 6A–D). From the four different fixation methods, glutaraldehyde, with a secondary osmium tetroxide fix, produced SEM images with the clearest definition of features (Figure 6D–F). Micro-CT scans produced three-dimensional representations of the microsphere tissue disk (Figure 6G). By computationally taking a thin slice of this model, collagen could be visualised throughout the microspheres, forming this tissue disk, with a thicker layer of collagen surrounding the microsphere tissue disk (Figure 6H).

## 3. Discussion

Various production methods and applications of polyHIPE microspheres, also called polyHIPE beads [25] or macro-porous polymer beads [26], have been increasingly reported in the last 20 years, as highlighted in a number of excellent reviews [27,28,29]. The earliest studies on making polyHIPE microspheres were reported in 2002, when Zhang et al. [30] produced monodisperse acrylamide polyHIPE microspheres via sedimentation polymerisation, a method for making spherical polymer microspheres was developed by Ruckenstein et al. [31], and in the same year, Desforges et al. [25] reported the production of monodisperse styrene/divinyl benzene-based polyHIPE microspheres via a suspension-based droplet setup. In 2013, Gokmen et al. reported a continuous production system via a (micro)fluidic system for production of ethylene glycol dimethacrylate (EGDMA)-based beads, in a similar experimental setup as that used in this study [32]. This setup was recently used by Ferrer et al. [26] for scalable production of EGDMA-based microspheres. Alternative routes for production have been reported, including a pipette tip-based microfluidic setup [33] and an electric field-assisted microfluidic platform [34]. Since their inception, these microspheres have been highlighted as potential injectable scaffolds for tissue engineering. To achieve this application, microspheres of biocompatible or biodegradable materials have been produced, in particular protein-laden EGDMA microspheres for bone tissue engineering were reported by the Cosgriff-Hernandez group [35,36]. Paterson et al. [37] reported the use of both polydisperse (produced by a controlled stirred-tank reactor (CSTR)) and monodisperse (produced by a T-junction fluidic) non-degradable (isobornyl acrylate/ethyl hexyl acrylate) polyHIPE microspheres as scaffolds for bone tissue engineering. The microspheres had the correct porosity to observe mesenchymal cell ingrowth and cell-laden microspheres invoked an angiogenic response in a chorioallantoic membrane (CAM) assay. Costatini et al. highlighted the use of monodisperse alginate/chitosan microspheres [34] for 3D culture of hMSCs. Additionally, the production of thiol-ene microspheres for biological applications has been reported by Kramer et al. [38], whereby these polydisperse microspheres were produced via a CSTR process. The same group also highlighted the use of macroscopic disk thiol-ene polyHIPE scaffolds as potential scaffolds for cartilage repair [39] since they showed good chondrocyte ingrowth and cartilage production in in vitro studies. The clinical applications of these porous microspheres can also be compared and contrasted with non-porous microspheres. Degradable non-porous microspheres are often used in clinical applications for drug delivery or as injectable materials for embolization agents [40], while porous microspheres enable cell ingrowth and tissue integration and are more applicable for injectable tissue engineering solutions.

In this study, we explored the use of PGS-based polyHIPE microspheres for injectable scaffolds for cartilage repair. The produced microspheres underwent tests in three key areas. Firstly, we studied whether these novel PGSm porous microspheres could be made in a reproducible manner with appropriate mechanical properties. Secondly, we assessed whether the material and scaffold are suitable for cartilage tissue engineering through in vitro analysis with primary chondrocytes. Thirdly, we found suitable techniques to assess and image the tissue-engineered constructs to determine the suitability of the microspheres.

### 3.1. PGSm polyHIPE, Microspheres, and Mechanical Analysis

Through the systematic modulation of synthesis parameters, both the bulk polyHIPE and the microsphere properties can be tuned. Experiments were performed during poly-HIPE synthesis to determine the ideal parameters for the synthesis of a stable PGSm HIPE. By varying the mixing speed and water content, differences were observed in the porosity, interconnectivity, and pore size. A mixing speed of 450 rpm and a water content of 0.82 were chosen as they provided adequate spread of homogenous interconnected pores between 10 and 50 µm, interconnects of approximately 6–10 μm (Figure 2A) and an average stiffness of 4 MPa. During microsphere synthesis, variations in needle size, syringe pump speed, water flow speed and the viscosity of water/media caused changes in microsphere dimensions, geometry and surface porosity. For the optimisation, three different size needles (30 G, 23 G and 15 G) and dispensing flow rates were explored. The largest needle diameter showed the largest polydispersity in microspheres, which decreased with the decreasing needle diameter. The polydispersity also increased with the syringe pump injection rate. For the remainder of the study, we selected an injection rate of 0.4 mL/h to produce microspheres with a size distribution of 465 ± 158 µm, with 82% porosity and a surfactant-to-polymer ratio of 20%. These sizes and injection speeds are in line with other studies. Indeed, Ferrer et al. [26] optimised EGDMA microsphere production, whereby they opted for a 22 G needle with an inner diameter of 0.41 mm and with an injection flow rate of 5.4 mL/h. This study produced microspheres of 554 ± 103 µm, with 80% porosity and a 15% surfactant-to-polymer ratio. Moglia and Whitely et al. [35,36] used smaller gauge needles in their experimental design (27 G and 30 G) and produced microspheres of 818 ± 61 µm, with a 27 G 0.21 mm inner diameter needle and an injection flow rate of 0.4 mL/h, for an EGDMA emulsion with a 30% surfactant-to-polymer ratio and 75% porosity. This reduced to 391 ± 60 µm with a 30 G 0.159 mm inner diameter needle and an injection flow rate of 0.8 mL/h. Our study indicated that there is an upper limit to the needle diameter to produce narrow size distributions. The use of a 15 G (1.372 mm inner diameter) syringe needle produced very broad size distributions, comparable with CSTR experiments [37]. As highlighted by Ferrer et al. [26], the flow behaviour around the needle tip is very likely laminar, but small perturbations produced by the needle tip (also called Karman vortices) [41] will increase the bead size distribution, providing an explanation as to why larger needle tips produce wider size distributions.

To produce the microspheres, the 23 G needle diameter was chosen as it produced an even spread of heterogeneously sized microspheres. The microspheres had a size distribution between 200 and 800 µm, and the upper boundary of the size allows them to be delivered via an arthroscopic cannula (typical diameter > 2 mm). Additionally, a heterogeneous size distribution allows for closer packing of the spheres once delivered. Homogeneously sized spheres have a maximum packing density of 74% [42]. This packing density does increase with the use of bimodal and heterogeneously sized spheres [43,44]. This occurs when spheres of differing sizes find the vacant spaces between the homogenously sized microspheres.

Additional parameter changes could improve the microsphere size distribution but could also potentially alter the stability and surface porosity of the polyHIPE microbeads, in particular the carrier fluid can be changed from pure water to a higher-viscosity liquid (e.g., in [26,36], a 3% poly(vinyl alcohol) solution is used as the carrier phase). A further determination of the fluid dynamics within the fluidic system joint with modelling could yield a predictive model of the polyHIPE microspheres’ size distribution within this system.

PGSm samples did not show any significant degradation over 40 days in PBS; therefore, enzymatic degradation studies were performed. Enzymatic degradation studies showed that PGSm polyHIPE degraded faster in the presence of lipase than PGSm samples, and this was expected as the PGSm polyHIPE material has a significantly higher surface area-to-volume ratio. Lipase is known to be secreted by macrophages thought to be present during cartilage repair; therefore, lipase enzymatic studies are considered a relevant in vitro degradation model [45]. The PGSm polyHIPE is biodegradable, and therefore has the utility to be used as a sacrificial carrier scaffold for cells; however, it is still important to have a similar modulus to that of the native tissue, as it has been well-documented that substrate mechanical stimuli can directly affect chondrocyte cultures [46]. The bulk compressive modulus of the polyHIPE material from the HIPE used for microsphere production was 4 MPa. Dynamic compressive testing results in the literature of human articular cartilage at 20 ms after loading report modulus values of 4.4–27.2 MPa [47]. Whilst this dynamic test is not directly comparable, it provides a guidance number for appropriate modulus values, with the bulk material having a similar modulus to the lower-boundary value for cartilage, as stated in the literature. Mechanical testing of individual porous PGSm microspheres proved problematic due to the size of the microspheres. Following three weeks of chondrocyte culture, the microspheres formed a microsphere tissue disk, with chondrocytes building collagen fibres and ECM components between the microspheres. Compressive mechanical tests were performed on the microsphere tissue disks in an unconfined manner, whilst wet, allowing for compression similar to native cartilage. Chondrocytes were cultured for three weeks, which is considered not a long enough period for fully mature tissue cultures; however, the microsphere tissue disks showed considerable strength and maintained their collective disk shape throughout the compression testing. The microsphere tissue disk had a modulus value of 30.3 MPa, similar to the 4.4–27 MPa found for native tissue in the literature [47]. It was also evident that the compressive modulus of the microsphere tissue disk was determined to be almost a factor of 10 higher than that of the bulk PGSm polyHIPE. Enzymatic degradation studies also showed a percentage mass loss of 27% after nine days, and it can therefore be postulated that a significant amount of the PGSm microspheres had already degraded after three weeks. This indicates that the ECM and collagen fibres created by the chondrocytes are mostly responsible for the strength and the microsphere tissue disks.

### 3.2. In Vitro Analysis of Tissue-Engineered Cartilage Scaffold

Assessing whether the scaffold/microspheres were appropriate for cartilage repair in vitro broadly covered five areas: chondrocyte attachment and survival, migration and ingress into the scaffolds, ability to produce the ECM, presence of GaGs and a confirmation of the presence of collagen-II.

Primary chondrocytes were cultured within PGSm polyHIPE microsphere scaffolds for a duration of three weeks. Prior to experimentation, wells were coated in agarose, a coating known to provide a non-adhesive 2D coating for cell attachment [48]. Chondrocytes did not attach to the bottom of the wells, which provided an increased probability for chondrocytes to attach to the microsphere scaffolds. During the experimentation, there were clear differences noted macroscopically between wells containing microspheres seeded with chondrocytes and wells containing cell-free microspheres. Individual microspheres seeded with chondrocytes had joined together to make one collective microsphere tissue disk, which was surrounded by a dense ECM. The results confirmed cell metabolic activity in wells with chondrocyte-seeded microspheres (Figure 3). The metabolic activity of the chondrocyte-seeded microspheres exceeded the negative control (cell-free microspheres) and cells seeded on agarose without microspheres, indicating cell attachment and survival within the PGSm porous microspheres over the three-week period. The metabolic activity within wells containing chondrocyte-seeded microspheres marginally decreased at each time point. This may be understood as natural levels of cell death related to the use of differentiated primary chondrocytes, which are less likely to proliferate. Furthermore, whilst the resazurin assay is a gentle, non-destructive procedure, there will always be a small amount of cell death related to stresses imparted on the cells via this procedure. The result of the cell viability assay indicated attachment and survival of chondrocytes on the PGSm polyHIPE microporous microspheres.

One limitation of polyHIPE and other porous scaffolds can be the slow speed at which the cells migrate and ingress into the scaffold, particularly in larger scaffolds. Approaches such as injection cell seeding can force cell ingress into large scaffolds, however, this can also introduce unwanted shear stresses on the cells. Another common limitation of large bulk porous scaffolds is the difficulty in tailoring the shape of the scaffold. The use of smaller polyHIPE microspheres allows for easier cell ingress into the scaffold and the opportunity to use the microspheres together to create larger scaffolds. Cell ingress into a scaffold can be an indicator of whether a scaffold is suited for use in tissue engineering, and this requires an interconnected porous microstructure with appropriate pore sizes and interconnectivity, enabling the culture of cells and nutrient exchange. The highly porous microspheres contain an intricate 3D microenvironment, acting as a scaffold supporting chondrocyte culture. The 3D microenvironments have been shown to be extremely important in tissue engineering of cartilage, where dedifferentiation can occur in 2D cultures [15].

Following three weeks of culture, histological analysis was performed to understand the composition of the three-dimensional tissue microsphere disk. H&E stains allowed the visualisation of cell nuclei (Haematoxylin) and extracellular matrix/cytoplasm (Eosin) [49]. H&E images (Figure 4) showed chondrocyte cells attached to the porous microspheres, both outside and within the microspheres up to depths of 200–400 µm. This compares well with the reported chondrocyte ingress in thiol-ene polyHIPE disks of 300 µm [39]. Collagen fibrils were observed through the specific staining of the ECM using Picro sirius red. The results showed collagen fibril formation surrounding and penetrating the porous microspheres, binding them together in the tissue microsphere disk. Glycosaminoglycans (GaGs) are another major component of the cartilage ECM secreted by hypertrophic chondrocytes and are an indication of a more mature cartilage-like chondrocyte culture [50]. Toluidine blue has a high binding affinity to sulphated GaGs, and the high concentration of the GaG-bound dye and the metachromatic nature of the dye caused it to appear purple [51,52,53]. The presence of GaGs was indicated from the purpling of the toluidine blue stain used during histological analysis. The DMB assay (Figure 4) confirmed the presence of GaGs within the chondrocyte-seeded microspheres. The presence of GaGs is one indicator that the microsphere tissue disks have formed a cartilage-like tissue, while another indicator is the presence of collagen, primarily collagen-II.

Collagen-II is the principal component of cartilage and is predominantly found in articular and hyaline cartilage, with 60% of the dry weight of cartilage as collagen, and of that, 90–95% is collagen-II [3,54]. A small percentage of other collagen types is also present, including collagen-I and collagen-III [55]. Studies have suggested that the hue of the imaged fibres present when using polarised light microscopy with a Picro sirius red stain may be related to the type of collagen fibre. Green fibres are understood as collagen-III and thicker red fibres as collagen-I [56]. This initial idea has since been revisited in the literature, and more recent studies show a relationship between the hue of the fibres and maturity, with juvenile thin fibres fluorescing as green, and more mature fibres as red [57,58]. Polarised light images of the Picro sirius red-stained microsphere tissue disk showed both red and green fluorescent collagen fibres, indicating the presence of both mature and more juvenile collagen fibres.

Immunohistochemistry labelling of microsphere tissue disks with anti-collagen-II antibodies showed collagen-II fibres completely covering and connecting individual microspheres (Figure 4). The presence of collagen-II, which is predominant in cartilage, is a good indicator of the formation of cartilage tissue.

### 3.3. Imaging Techniques: 2D, Internal, and 3D Representations

The tissue-engineered microsphere disk’s irregular three-dimensional shape and macro-size proved difficult to be imaged for microscopic analysis. Traditional histological techniques did work, however, slicing with the microtome often led to damage of the porous polyHIPE microspheres. Distortion of the microspheres with histology did not allow for a complete representation of the 3D tissue. The microsphere tissue disk was also IHC-labelled and imaged with a confocal microscope. Cell nuclei were stained by DAPI (blue), and the F-actin was stained by phalloidin (red) (Figure 5). Taking a Z-stack of the microsphere tissue disk enabled a non-destructive representation of the 3D scaffold. This allowed for the determination of individual cells and collagen fibres throughout the surface of the tissue microsphere disk. Using the autofluorescence of the polymer (green) allowed for high-resolution images, showing the interaction of the chondrocytes and the ECM with the porous microspheres (Figure 5). One limitation, however, was the depth of penetration achieved through confocal microscopy. Even with the additional benefit of using two-photon microscopy, confocal microscopy was limited in the depths achievable, and complete 3D constructs were not possible.

An alternative method used to visualise the complete three-dimensional microsphere tissue disk surface was through SEM. Several fixation techniques were explored, and glutaraldehyde fixation with an osmium tetroxide second fix provided the best SEM results. Individual chondrocytes were visible within the collagen/ECM surrounding the microspheres. Osmium tetroxide stained the cell membrane, which led to easy identification of cells because of their increased brightness in the SEM, and contrast of cells embedded within the ECM was possible with the use of false colouring (Figure 6). Chondrocytes interspersed within the dense ECM appeared similar in structure and comparable to that of hyaline cartilage. Although SEM provided a representation of the complete three-dimensional model, it only allowed for the visualisation of the surface of the microsphere tissue disk.

Micro-CT was later explored to create a complete three-dimensional reconstruction of the microsphere tissue disk. Micro-CT is a technique more often implemented for use with harder bone. Traditionally, the imaging of cartilage/tissue-engineered cartilage using micro-CT was difficult due to the weak signal from the cartilage [59]. An osmium tetroxide fix was used in this study to provide better contrast from the soft tissue. Whilst there is little and perhaps no evidence of this technique being used for cartilage tissue micro-CT imaging, osmium tetroxide has been used to stain many soft tissues. It is used as the electron-binding energies are well-suited for strong X-ray absorption. Micro-CT scans were able to accurately image the osmium tetroxide-stained soft microsphere tissue disks and allowed for the complete 3D modelling of the entire construct. Individual slices/sections could also be viewed from the models created, providing an insight into both the complete model and the internal structures of these microsphere tissue disks. Collagen fibres could be seen surrounding and penetrating individual polymer microspheres, highlighting the potential use of micro-CT for imaging three-dimensional tissue-engineered cartilage, through fixation with osmium tetroxide. Unlike confocal microscopy, micro-CT allows for complete penetration throughout the entire scaffold; however, confocal microscopy offers a far higher resolution and therefore a better representation of the available data, with the benefit of specific IHC staining. SEM can also show high-resolution images, with false colours highlighting individual cells; however, only the surface of the tissue is visible. By using a combination of these techniques, it was possible to build a more complete picture of this microsphere tissue disk, with high-resolution 2D and 3D imaging, specific staining and internal components visualised.

The results reported in this study are complementary to emerging research on PGS-based scaffolds for cartilage repair. Hollister’s group first reported a fused-filament-fabricated (FFF) PGS scaffold for cartilage repair in 2010, and compared it to polycaprolactone (PCL) and poly-(1,8 octanediol-co-citrate) (POC) in 2015 [17,18]. They concluded in the latter study that POC is more favourable to use for cartilage repair and that both PCL and PGS have a tendency to induce ossification of the grown tissue. This behaviour might be influenced by the particular format of the biomaterial, and more microporous materials might provide a more natural 3D environment for the chondrocytes. This was exemplified by a recent study using a polyester-based polyHIPE scaffold, showing excellent chondrocyte ingrowth (up to 300 µm) and cartilage production (assessed via collagen-II immunohistochemistry) [39]. The current study indicated a similar result with PGS microspheres, where both the chondrocyte ingrowth and the high GAG/collagen-II content indicated preservation of the chondrogenic phenotype. Additionally, Silva et al. highlights the use of an electro-spun PGS/PCL composite nano-fibre mat with inclusion of a small-molecule compound (Kartogenin) to stimulate chondrogenic differentiation of hBMSCs. These nano-fibre mats are suggested to be used in conjunction with MSCs as a therapy to repair defects in the superficial zone of articular cartilage. Of note is that a PGS-co-PEG copolymer-foamed microporous construct (20–40 µm pore size) has recently been investigated by Lin et al. [22] and showed excellent regenerative potential and integration after 12 weeks in vivo when implanted in a New Zealand white rabbit full-thickness osteochondral defect. These studies indicate that materials with 20–100 µm pores are excellent 3D microenvironments for chondrocyte ingrowth, without noticeable ossification. Additionally, the literature indicates that inclusion of chondrogenic factors can further enhance the efficiency of these scaffolds, which will be a future direction of research.

## 4. Materials and Methods

### 4.1. Emulsion Synthesis/PGSm HIPE Production

PGSm was synthesised in accordance with methodologies described in [23]. In short, equimolar amounts of sebacis acid and glycerol were reacted at 120 °C in a nitrogen atmosphere for 24 h, and subsequently in a vacuum for 24 h. After 48 h, the prepolymer was cooled in an ice bath and an equal volume of dichloromethane was added to dissolve the prepolymer. Methacrylic anhydride was slowly added at a 1.00 mol/mol ratio of hydroxyl groups in PGS, and an equimolar amount of trimethylamine was added. The reaction vessel was left stirring for 24 h, while being allowed to return to room temperature. The product was purified by washing in methanol and precipitation at −80 °C, acid-washed in an aqueous solution of hydrochloric acid and further dried on magnesium sulphate, and then the remaining solvent was removed via rotary evaporation.

### 4.2. Microsphere Production

A close-circuit system comprised of a peristaltic pump (Masterflex L/S, ColeParmer, Saint Neots, UK), syringe pump (World Precision Instruments, Hitchin, UK), ultraviolet (UV) curing chamber, collector and H_2_O reservoir was used to produce the microspheres (see schematic in Figure 1B). PGSm emulsion was contained in a 10 mL syringe, attached to a syringe pump. The needle of the syringe was inserted into the fluidic tubing, with the peristaltic pump flowing water across the tip of the syringe (perpendicular). As the syringe pump expelled the emulsion into the tubing, droplets of the emulsion were carried away by the water flow (as a carrier fluid), creating a water-in-oil-in-water system. The emulsion was passed through a curing chamber, where it was irradiated with UV light to crosslink the porous polymer microspheres. The microspheres were collected into a cell strainer, with excess water falling through the strainer and into the reservoir. Properties of the microspheres were varied by adjusting the needle size, flow rate and the syringe pump rate. The needle outer diameters explored were 0.312 mm (30 G, 0.159 mm inner diameter), 0.642 mm (23 G, 0.337 mm inner diameter) and 1.829 mm (15 G, 1.372 mm inner diameter), the injection flow rates were between 0.1 mL min^−1^ and 0.7 mL min^−1^ and the peristaltic pump was maintained at 450 rpm for water flow. Following the initial tests, microspheres were produced with a needle diameter of 0.642 mm and a syringe flow rate of 0.4 mL min^−1^ for all further experimentation. Crosslinked polyHIPE microspheres were then introduced to methanol and cycled within a Soxhlet extractor overnight, to remove any un-crosslinked polymer and to allow for the removal of water from within the pores. Any methanol remaining within the pores was evaporated away at room temperature or under a vacuum.

### 4.3. Mechanical Compression Test of PGSm polyHIPE

Emulsions were synthesised as above and poured into moulds, and the polymer was fully crosslinked in the presence of UV light. Flat sheets of PGSm polyHIPE were photo-crosslinked at a 5 mm thickness. Circular samples of 6 mm in diameter were cut from the sheet using a biopsy punch. The polyHIPE samples were soaked in methanol and allowed to dry, to ensure the removal of water from within the pores. The polyHIPE disks were transferred to the mechanical testing machine for analysis. Mechanical parameters were assessed in compression using a Hounsfield mechanical tensometer (H100ks, Hounsfield, Redhill, UK) and a 10 N load cell (the1-10N). The compression test was set at a rate of 0.25 mm/min, with a maximum load of 10 N. The area and depth of the polyHIPE disks were used to calculate the compressive modulus value of the samples.

### 4.4. Enzymatic Degradation of PGSm polyHIPE

Samples were punched from a flat sheet of PGSm polyHIPE and solid PGSm. Enzymatic degradation studies were performed in phosphate-buffered saline (PBS) with a physiological pH. Samples were prepared with a diameter of 6 mm and a thickness of 3 mm. The disks were placed in a 24-well plate. Lipase from *Thermomyces lanuginosus* (100,000 U/g Sigma-Aldrich, Poole, UK), at a concentration of 2000 U/mL in PBS, was added to each well. The well plate was placed on an orbital shaker and incubated at 37 °C for the duration of the experiment. The PGSm disks were weighed before the lipase solution was added, at regular intervals throughout the experiment, with excess solution shaken off at the end of the experiment once dry. The size of the samples, and the change in weight over three days, were used to calculate the rate of degradation of the PGSm disks.

### 4.5. Chondrocyte Cell Isolation and Culture

Cells were isolated and provided by SL, Sheffield. Metacarpophalangeal joints were obtained from a local abattoir from skeletally mature beef cattle (18–24 months) within 6 hours of slaughter. Joints were sprayed with 70% IMS and 10% Trigene. To further minimise infection risks, all areas of the joint that were not being worked on were wrapped in foil. A post-mortem blade was used to remove the skin from the joints. Bovine metacarpophalangeal joints (the third metacarpal on the hoof) were used, and the skinned joints were moved to a class-2 laminar air cabinet, where all following procedures were aseptically performed. A sterile scalpel was used to pry open the joint, and using the scalpel and tweezers, articular cartilage was removed from the surface of the joints and placed into Ca^2+^/Mg^2+^-free PBS.

Articular cartilage pieces were removed from PBS, incubated in 2.5% (*w*/*v*) trypsin at 37 °C for 30 min and then washed in fresh PBS. High-glucose DMEM (Dulbecco’s Modified Eagle Medium), containing 1000 units/mL penicillin, 1000 µg/mL streptomycin, 10% FCS (foetal calf serum) and 2 mg/mL bacterial collagenase, was incubated with the cartilage pieces on an orbital shaker in a 37 °C incubator and shaken at 30 rotations per minute for 18 h. After tissue digestion, the cell-containing medium was transferred into centrifuge tubes and centrifuged at 1000 rpm, 192 g, for 10 minutes. The centrifugation resulted in a pellet of chondrocytes; the supernatant was removed, and the pellet was resuspended in PBS. Cells were centrifuged in this manner a further two times. The resultant cell pellet was then resuspended in high-glucose DMEM, and a haemocytometer was used to calculate cell numbers. A monolayer culture of chondrocytes was then setup in T75 flasks, with basic medium (high-glucose DMEM containing 1000 units/mL penicillin, 1000 µg/mL streptomycin, non-essential amino acids and 10 mM HEPES buffer, pH 7.4, and 10% *v*/*v* FCS). All cell cultures were incubated at 37 °C in a humidified atmosphere of 95% air and 5% CO_2_. All experiments were performed using passage 1 cells to minimise any dedifferentiation from the 2D culture. Cells were passaged in a standard manner using 0.05% trypsin and 0.02% EDTA at 37 °C for 5 min.

### 4.6. Chondrocyte Seeding

Porous PGSm microspheres were centrifuged and redispersed in 70% ethanol for sterilisation. PBS and media washes followed in a similar manner, and centrifugation allowed for liquids to rapidly ingress into the porous material. A 96-well plate was prepared by adding 15 µL of warm agarose solution (2%) to each well. As the agarose cooled, it solidified, creating a flat coating on the bottom of the wells, and this ensured that the chondrocytes would not adhere to the bottom of the well. Microspheres were dispersed in the medium, and 200 µL of microsphere-containing medium (between 25 and 50 microspheres) was added to the wells in a 96-well plate.

Chondrocytes were dispersed in chondrocyte medium (high-glucose DMEM containing 100 units/mL penicillin, 100 µg/mL streptomycin, 10% FCS and 10 ng/mL FGF), counted and plated in T75 flasks for culture. Cells were used at passage 1. Then, 50,000 cells were added to each of the wells, either containing PGSm microspheres or controls, and the plate was placed on an orbital shaker inside of an incubator for a period of 48 h to allow the cells to evenly attach to the microspheres. After 48 h, differentiation medium was added to the wells (chondrocyte medium with 50 µg/mL L-ascorbic acid, 1 µg/mL insulin), and this medium was refreshed every 3–4 days. After 48 h, cells began to produce the ECM, visible between adjoining microspheres.

### 4.7. Resazurin/Alamar Blue Assay

A cell viability assay using resazurin/Alamar blue was conducted on days 2, 7, 14 and 21. Microspheres seeded with cells, cell-free microspheres and cells cultured on agarose were analysed. A stock solution of Alamar blue was made for all experiments from 12.6 mg of resazurin dissolved in 50 mL of PBS. The medium was removed from the cell culture well and replaced with Alamar blue medium solution (1:10, Alamar blue stock solution to cell medium). The well plate was returned to the incubator, and after 4 h, a minimum of 3 samples were taken and read using a fluorescent plate reader (λ_Ex_/λ_Em_, 560/590 nm). Wells were gently washed with medium and then fresh medium was added to the well plate and returned to the incubator.

### 4.8. Dimethylmethylene Blue/GaG Assay

After three weeks of culture, a dimethylmethylene blue (DMB) assay was performed to quantify the presence of sulphated GaGs. The DMB assay was based on the method described by Farndale et al. [24]. Papain digestion solution (150 µL) (200 mM PBS containing 1 mM EDTA, 0.05% *w*/*v* papain, 0.96% *w*/*v* n-acetyl cysteine, pH 6.8) was added to microspheres, microsphere tissue disks and the medium in 0.5 mL Eppendorf tubes. Samples were incubated at 60 °C for 24 h. DMB (dimethylmethylene blue) stock solution (0.008 g DMB, 1.52 g glycine, 1.185 g NaCl, 500 mL H_2_O) was mixed in a lightproof bottle and stirred for 2 h. The DMB stock solution pH was adjusted to 3.0 with hydrochloric acid. Chondroitin sulphate stock, used for a standard curve, was diluted to 50 µg/mL in distilled water. Chondroitin sulphate calibrations (50 µL) were then used from 0 to 50 µg/mL and added to a 96-well plate to create a calibration curve. A water blank was added to the 96-well plate and 50 µL of digestion solution from the samples was added to the well plate, too. Using a multichannel pipette, 250 µL of DMB solution was added to all samples, calibrations and a reagent blank. The plate was then immediately measured in a plate reader at an optical density (OD) of 525 nm.

### 4.9. Cryosectioning

Microsphere tissue disks were fixed in 3.7% formaldehyde for 25 min and washed twice in PBS. The disks were placed into square plastic cryo-moulds and OCT medium (Agar Scientific, Stansted, UK) was poured both under and over the disk. The disk was properly aligned within the cryo-mould, and the mould was gently introduced to a small pool of liquid nitrogen for periods of 1–2 s. This was enough time to freeze the sample in OCT medium, without producing cracks. Once frozen, the block was placed in a cryotome and sliced. Slices between 6 and 15 µm were attached to Superfrost microscope slides (ThermoFisher Scientific, Horsham, UK). As the slides were brought to room temperature, the OCT medium melted, leaving the sample on the microscope slide.

### 4.10. Histochemical Staining

Haematoxylin and Eosin (H&E): Samples were immersed in xylene for 30 s, 100% ethanol (EtOH) for 30 s, 70% EtOH for 30 s and distilled water for 30 s. Sample slides were then immersed in Haematoxylin for 3 min, washed in running tap water for 5 min and then immersed in Eosin solution for 1 min. Slides were then washed in 100% EtOH for 5 min, DPX mountant was added and a cover slip was secured, covering the sample.

Picro sirius Red: Samples were dipped in 70% EtOH, rinsed in distilled water and placed in Picro sirius red solution (0.5 g sirius red/direct red 80, in 500 mL of saturated aqueous picric acid) for 60 min. Slides were then placed in acidified water for 5 min (5 mL acetic acid in 1 L distilled water). Then, slides were placed in 100% EtOH for 5 min, DPX mountant was added and a cover slip was secured, covering the sample.

Toluidine blue: A working solution was made from 5 mL of toluidine blue stock solution (1 g of toluidine blue O (Sigma-Aldrich) in 100 mL of 70% EtOH) and 45 mL of 1% sodium chloride, pH 2.3. Samples were dipped in 70% EtOH, then in distilled water, and were then placed in the toluidine blue working solution for 6 min. Slides were then washed in distilled water for 3 min, dried, DPX mountant was added and a cover slip was secured, covering the sample.

### 4.11. Immunofluorescence

Collagen-II antibody: Samples were fixed for 25 min. The primary antibody, anti-collagen-II antibody (Abcam, Trumpington, UK), was used at a titre of 1:100 in ICC buffer and left refrigerated overnight. The secondary antibody, Alexa 546 goat anti-rabbit IgG (λ_Ex_/λ_Em_, 556/573 nm max, ThermoFisher Scientific, Waltham, MA, USA), at a titre of 1:100 in ICC buffer, was added at room temperature for 2 h. The samples were then washed and submerged in PBS for imaging.

DAPI and phalloidin: Once fixed and washed, phalloidin-TRITC (tetramethylrhodamine) was added at a titre of 1:1000 in PBS (λ_Ex_/λ_Em_, 540/565 nm max, ThermoFisher Scientific, USA) for 30 min at room temperature. The solution was removed, and the sample was washed twice with PBS. DAPI (4′,6-diamidino-2-phenylindole) was added at a titre of 1:1000 in PBS for 10 min (λ_Ex_/λ_Em_, 350/470 nm max, ThermoFisher Scientific, Waltham, MA, USA), and the sample was then washed and submerged in PBS for imaging.

### 4.12. Scanning Electron Microscopy (SEM) and Micro-CT Preparation

Samples were freeze-dried before imaging via SEM to maintain the internal structure. Samples not containing cells were dried and gold-coated using a gold sputter coater and imaged using a Phillips XL-20 SEM. Unless otherwise stated, samples containing cells were rinsed in PBS and then fixed in glutaraldehyde (2.5% in PBS buffer) for 1 h. Samples were washed in PBS for 15 min, then in distilled water for 5 min. The samples were introduced to increasing concentrations of EtOH in distilled water (35%, 60%, 80%, 90%, 100%) for 15 min each. A 1:1 solution of EtOH and HMDS was introduced to the samples for 1 h inside a fume cupboard. Samples were then introduced to 2 100% HMDS washes for 5 min each. HMDS was removed and samples were air-dried and gold-coated. Samples to be analysed with micro-CT were rinsed in PBS and fixed in glutaraldehyde, as above. The samples underwent a second fix and were rinsed in PBS and introduced to an aqueous solution of 2% osmium tetroxide for 2 h. The samples were then analysed using a Skyscan 1272 high-resolution micro-CT.

### 4.13. Compressive Mechanical Analysis of Chondrocyte Disks

Following three weeks of culture, microsphere tissue disks were removed from culture plates and excess medium was shaken off. Disks were then photographed from above beside a scale to calculate the area of the disks using Image J (version 1.53t, National Institutes of Health, Bethesda, MD, USA). The area and depth of the disks were used in calculations for the compressive modulus of the tissue disks. The disks were placed between the two high-density polymer supports on the compression testing machine (Zwick, ZwickiLine Z0.5 up to Z2.5), loaded with a 500 N load cell, then the disks were compressed at a rate of 0.1 mm/s. The microsphere tissue disks remained intact throughout the experimentation.

## 5. Conclusions

This study assessed novel porous PGSm polyHIPE microspheres for use in cartilage tissue engineering. HIPE and microsphere synthesis techniques were successfully developed to synthesise reproducible, novel PGSm porous microspheres, with the bulk material and the microsphere tissue disk having compressive modulus values similar to that of native tissue. Three-week primary chondrocyte cultures demonstrated that the material and microspheres were a highly effective three-dimensional scaffold for chondrocyte culture. Chondrocytes attached to the microsphere scaffolds, produced an extended network of a dense ECM and created a strong, combined microsphere tissue disk with a high GaG/collagen-II content. Various imaging analyses showed the tissue-engineered microsphere tissue disk, with chondrocytes interspersed within a dense network of the ECM, having a similar structure to that of cartilage. Collagen fibres completely covered the samples, with the presence of GaGs and collagen-II confirmed, both of which are major components of the cartilage ECM, indicating the formation of cartilage-like tissue. The tissue microsphere disks were imaged using a variety of different techniques, including with the use of osmium tetroxide to enhance the attenuation of X-rays in micro-CT, to produce complete three-dimensional models of the microsphere tissue disk with increased contrast.

In summary, (i) mechanical analysis indicated the scaffold’s suitability for cartilage tissue engineering, (ii) the imaging techniques provided methodologies for analysis and (iii) the culture of chondrocytes with a dense ECM creating a microsphere tissue disk with a high GaG/collagen-II content highlighted the utility of the microspheres for the delivery of cell-laden scaffolds via microsurgery for cartilage tissue engineering.

## Data Availability

The data generated in this study are available from the authors upon request.

## References

[1] Xu Y., Fan F., Kang N., Wang S., You J., Wang H., Zhang B. (2015). Tissue Engineering of Human Nasal Alar Cartilage Precisely by Using Three-Dimensional Printing. Plast. Reconstr. Surg..

[2] Liao H.T., Zheng R., Liu W., Zhang W.J., Cao Y., Zhou G. (2015). Prefabricated, Ear-Shaped Cartilage Tissue Engineering by Scaffold-Free Porcine Chondrocyte Membrane. Plast. Reconstr. Surg..

[3] Sophia Fox A.J., Bedi A., Rodeo S.A. (2009). The basic science of articular cartilage: Structure, composition, and function. Sports Health.

[4] Kessler M.W., Grande D.A. (2008). Tissue engineering and cartilage. Organogenesis.

[5] Makris E.A., Gomoll A.H., Malizos K.N., Hu J.C., Athanasiou K.A. (2014). Repair and tissue engineering techniques for articular cartilage. Nat. Rev. Rheumatol..

[6] Steadman J.R., Rodkey W.G., Briggs K.K., Rodrigo J.J. (1999). The microfracture technic in the management of complete cartilage defects in the knee joint. Orthopade.

[7] Bae D.K., Yoon K.H., Song S.J. (2006). Cartilage Healing After Microfracture in Osteoarthritic Knees. Arthrosc. J. Arthrosc. Relat. Surg..

[8] Breinan H.A., Martin S.D., Hsu H.-P., Spector M. (2000). Healing of canine articular cartilage defects treated with microfracture, a type-II collagen matrix, or cultured autologous chondrocytes. J. Orthop. Res..

[9] Kreuz P.C., Steinwachs M.R., Erggelet C., Krause S.J., Konrad G., Uhl M., Südkamp N. (2006). Results after microfracture of full-thickness chondral defects in different compartments in the knee. Osteoarthr. Cartil..

[10] Chen H., Sun J., Hoemann C.D., Lascau-Coman V., Ouyang W., McKee M.D., Shive M.S., Buschmann M.D. (2009). Drilling and microfracture lead to different bone structure and necrosis during bone-marrow stimulation for cartilage repair. J. Orthop. Res..

[11] Peterson L., Vasiliadis H.S., Brittberg M., Lindahl A. (2010). Autologous Chondrocyte Implantation. Am. J. Sports Med..

[12] Bartlett W., Skinner J.A., Gooding C.R., Carrington R.W.J., Flanagan A.M., Briggs T.W.R., Bentley G. (2005). Autologous chondrocyte implantation versus matrix-induced autologous chondrocyte implantation for osteochondral defects of the knee: A Prospective, Randomised Study. J. Bone Jt. Surg.-Br..

[13] Foldager C.B., Gomoll A.H., Lind M., Spector M. (2012). Cell Seeding Densities in Autologous Chondrocyte Implantation Techniques for Cartilage Repair. Cartilage.

[14] Caron M.M.J., Emans P.J., Coolsen M.M.E., Voss L., Surtel D.A.M., Cremers A., van Rhijn L.W., Welting T.J.M. (2012). Redifferentiation of dedifferentiated human articular chondrocytes: Comparison of 2D and 3D cultures. Osteoarthr. Cartil..

[15] Ossendorf C., Kaps C., Kreuz P.C., Burmester G.R., Sittinger M., Erggelet C. (2007). Treatment of posttraumatic and focal osteoarthritic cartilage defects of the knee with autologous polymer-based three-dimensional chondrocyte grafts: 2-year clinical results. Arthritis Res. Ther..

[16] Nesic D., Whiteside R., Brittberg M., Wendt D., Martin I., Mainilvarlet P. (2006). Cartilage tissue engineering for degenerative joint disease. Adv. Drug Deliv. Rev..

[17] Kemppainen J.M., Hollister S.J. (2010). Tailoring the mechanical properties of 3D-designed poly(glycerol sebacate) scaffolds for cartilage applications. J. Biomed. Mater. Res. Part A.

[18] Jeong C.G., Hollister S.J. (2010). A comparison of the influence of material on in vitro cartilage tissue engineering with PCL, PGS, and POC 3D scaffold architecture seeded with chondrocytes. Biomaterials.

[19] Patel A., Gaharwar A.K., Iviglia G., Zhang H., Mukundan S., Mihaila S.M., Demarchi D., Khademhosseini A. (2013). Highly elastomeric poly(glycerol sebacate)-co-poly(ethylene glycol) amphiphilic block copolymers. Biomaterials.

[20] Souza M.T., Tansaz S., Zanotto E.D., Boccaccini A.R. (2017). Bioactive Glass Fiber-Reinforced PGS Matrix Composites for Cartilage Regeneration. Materials.

[21] Silva J.C., Udangawa R.N., Chen J., Mancinelli C.D., Garrudo F.F.F., Mikael P.E., Cabral J.M.S., Ferreira F.C., Linhardt R.J. (2020). Kartogenin-loaded coaxial PGS/PCL aligned nanofibers for cartilage tissue engineering. Mater. Sci. Eng. C.

[22] Lin D., Cai B., Wang L., Cai L., Wang Z., Xie J., Lv Q., Yuan Y., Liu C., Shen S.G.F. (2020). A viscoelastic PEGylated poly(glycerol sebacate)-based bilayer scaffold for cartilage regeneration in full-thickness osteochondral defect. Biomaterials.

[23] Singh D., Harding A.J., Albadawi E., Boissonade F.M., Haycock J.W., Claeyssens F. (2018). Additive manufactured biodegradable poly(glycerol sebacate methacrylate) nerve guidance conduits. Acta Biomater..

[24] Farndale R.W., Sayers C.A., Barrett A.J. (1982). A direct spectrophotometric microassay for sulfated glycosaminoglycans in cartilage cultures. Connect. Tissue Res..

[25] Desforges A., Arpontet M., Deleuze H., Mondain-Monval O. (2002). Synthesis and functionalisation of polyHIPE^®®^ beads. React. Funct. Polym..

[26] Ferrer J., Jiang Q., Menner A., Bismarck A. (2022). An approach for the scalable production of macroporous polymer beads. J. Colloid Interface Sci..

[27] Zhang H., Cooper A.I. (2005). Synthesis and applications of emulsion-templated porous materials. Soft Matter.

[28] Gokmen M.T., Du Prez F.E. (2012). Porous polymer particles—A comprehensive guide to synthesis, characterization, functionalization and applications. Prog. Polym. Sci..

[29] Liu Y., Feng Y., Yao J. (2016). Recent advances in the direct fabrication of millimeter-sized hierarchical porous materials. RSC Adv..

[30] Zhang H., Cooper A.I. (2002). Synthesis of monodisperse emulsion-templated polymer beads by oil-in-water-in-oil (O/W/O) sedimentation polymerization. Chem. Mater..

[31] Ruckenstein E., Hong L. (1995). Sedimentation polymerization. Polymer.

[32] Gokmen M.T., Dereli B., De Geest B.G., Du Prez F.E. (2013). Complexity from Simplicity: Unique Polymer Capsules, Rods, Monoliths, and Liquid Marbles Prepared via HIPE in Microfluidics. Part. Part. Syst. Charact..

[33] Lapierre F., Cameron N.R., Zhu Y. (2015). Ready… set, flow: Simple fabrication of microdroplet generators and their use in the synthesis of PolyHIPE microspheres. J. Micromechanics Microengineering.

[34] Costantini M., Guzowski J., Żuk P.J., Mozetic P., De Panfilis S., Jaroszewicz J., Heljak M., Massimi M., Pierron M., Trombetta M. (2018). Electric Field Assisted Microfluidic Platform for Generation of Tailorable Porous Microbeads as Cell Carriers for Tissue Engineering. Adv. Funct. Mater..

[35] Moglia R., Whitely M., Brooks M., Robinson J., Pishko M., Cosgriff-Hernandez E. (2014). Solvent-Free Fabrication of polyHIPE Microspheres for Controlled Release of Growth Factors. Macromol. Rapid Commun..

[36] Whitely M., Rodriguez-Rivera G., Waldron C., Mohiuddin S., Cereceres S., Sears N., Ray N., Cosgriff-Hernandez E. (2019). Porous PolyHIPE microspheres for protein delivery from an injectable bone graft. Acta Biomater..

[37] Paterson T.E., Gigliobianco G., Sherborne C., Green N.H., Dugan J.M., MacNeil S., Reilly G.C., Claeyssens F. (2018). Porous microspheres support mesenchymal progenitor cell ingrowth and stimulate angiogenesis. APL Bioeng..

[38] Kramer S., Krajnc P. (2021). Hierarchically Porous Microspheres by Thiol-ene Photopolymerization of High Internal Phase Emulsions-in-Water Colloidal Systems. Polymers.

[39] Naranda J., Sušec M., Maver U., Gradišnik L., Gorenjak M., Vukasović A., Ivković A., Rupnik M.S., Vogrin M., Krajnc P. (2016). Polyester type polyHIPE scaffolds with an interconnected porous structure for cartilage regeneration. Sci. Rep..

[40] Yang X., Wang S., Zhang X., Ye C., Wang S., An X. (2022). Development of PVA-based microsphere as a potential embolization agent. Biomater. Adv..

[41] Von Kármán T. (2004). Aerodynamics: Selected Topics in the Light of Their Historical Development.

[42] Scott G.D., Kilgour D.M. (1969). The density of random close packing of spheres. J. Phys. D Appl. Phys..

[43] Bennett J.P., Smith J.D. (2012). Fundamentals of Refractory Technology.

[44] Knott G.M., Jackson T.L., Buckmaster J. (2001). Random Packing of Heterogeneous Propellants. AIAA J..

[45] Li M., Yin H., Yan Z., Li H., Wu J., Wang Y., Wei F., Tian G., Ning C., Li H. (2022). The immune microenvironment in cartilage injury and repair. Acta Biomater..

[46] Chen C., Xie J., Deng L., Yang L. (2014). Substrate Stiffness Together with Soluble Factors Affects Chondrocyte Mechanoresponses. ACS Appl. Mater. Interfaces.

[47] Shepherd D.E.T., Seedhom B.B. (1997). A technique for measuring the compressive modulus of articular cartilage under physiological loading rates with preliminary results. Proc. Inst. Mech. Eng. Part H J. Eng. Med..

[48] Svoronos A.A., Tejavibulya N., Schell J.Y., Shenoy V.B., Morgan J.R. (2014). Micro-mold design controls the 3D morphological evolution of self-assembling multicellular microtissues. Tissue Eng. Part A.

[49] Fischer A.H., Jacobson K.A., Rose J., Zeller R. (2008). Hematoxylin and eosin staining of tissue and cell sections. CSH Protoc..

[50] Jouls V., Bocquet J., Pujol J.-P., Brisset M., Loyau G., Glycosammoglycan P. (1985). Effect of asorbic acid on secreted proteoglycans from rabbit articular chondrocytes. FEBS Lett..

[51] Schmitz N., Laverty S., Kraus V.B., Aigner T. (2010). Basic methods in histopathology of joint tissues. Osteoarthr. Cartil..

[52] Sridharan G., Shankar A.A. (2012). Toluidine blue: A review of its chemistry and clinical utility. J. Oral Maxillofac. Pathol..

[53] Athanasiou K.A., Darling E.M., DuRaine G.D., Hari Reddi J.C.H.A. (2013). Articular Cartilage.

[54] Eyre D. (2002). Collagen of articular cartilage. Arthritis Res..

[55] Ricard-Blum S., Herbage D. (1985). The Different Types of Collagen Present in Cartilaginous Tissues. Biology of Invertebrate and Lower Vertebrate Collagens.

[56] Junqueira L.C.U., Cossermelli W., Brentani R. (1978). Differential Staining of Collagens Type I, II and III by Sirius Red and Polarization Microscopy. Archivum Histologicum Japonicum.

[57] Yassin F.E.-Z.E.-D., El-Dawela R., Kerim M. (2014). Picrosirius red staining assessment of collagen after dermal roller application: A minimally invasive percutaneous collagen induction therapy. Indian J. Dermatopathol. Diagn. Dermatol..

[58] Rich L., Whittaker P. (2005). Collagen and PICrosirius Red Staining: A Polarized Light Assessment of Fibrillar Hue and Spatial Distribution. Braz. J. Morphol. Sci..

[59] Mansour J.M., Welter J.F. (2013). Multimodal evaluation of tissue-engineered cartilage. J. Med. Biol. Eng..

